# Hospitalizations for Endocarditis and Associated Health Care Costs Among Persons with Diagnosed Drug Dependence — North Carolina, 2010–2015

**DOI:** 10.15585/mmwr.mm6622a1

**Published:** 2017-06-09

**Authors:** Aaron T. Fleischauer, Laura Ruhl, Sarah Rhea, Erin Barnes

**Affiliations:** ^1^Epidemiology Section, North Carolina Division of Public Health; ^2^Career Epidemiology Field Officer, Office of Public Health Preparedness and Response, CDC; ^3^Department of Preventive Medicine, University of North Carolina at Chapel Hill; ^4^Preventive Medicine Fellowship, CDC; ^5^Wake Forest University Medical Center, Winston-Salem, North Carolina.

Opioid dependence and overdose have increased to epidemic levels in the United States. The 2014 National Survey on Drug Use and Health estimated that 4.3 million persons were nonmedical users of prescription pain relievers (*1*). These users are 40 times more likely than the general population to use heroin or other injection drugs (*2*). Furthermore, CDC estimated a near quadrupling of heroin-related overdose deaths during 2002–2014 (*3*). Although overdose contributes most to drug-associated mortality, infectious complications of intravenous drug use constitute a major cause of morbidity leading to hospitalization (*4*). In addition to infections from hepatitis C virus (HCV) and human immunodeficiency virus (HIV), injecting drug users are at increased risk for acquiring invasive bacterial infections, including endocarditis (*5*,*6*). Evidence that hospitalizations for endocarditis are increasing in association with the current opioid epidemic exists (*7*–*9*). To examine trends in hospitalizations for endocarditis among persons in North Carolina with drug dependence during 2010–2015, data from the North Carolina Hospital Discharge database were analyzed. The incidence of hospital discharge diagnoses for drug dependence combined with endocarditis increased more than twelvefold from 0.2 to 2.7 per 100,000 persons per year over this 6-year period. Correspondingly, hospital costs for these patients increased eighteenfold, from $1.1 million in 2010 to $22.2 million in 2015. To reduce the risk for morbidity and mortality related to opioid-associated endocarditis, public health programs and health care systems should consider collaborating to implement syringe service programs, harm reduction strategies, and opioid treatment programs.

The North Carolina Hospital Discharge database (processed by Truven Health Analytics for the North Carolina State Center for Health Statistics) included discharge data from all 128 hospitals in North Carolina, accounting for approximately 1 million hospital admissions per year. Patients aged ≥18 years who were discharged with diagnosis codes (ninth and tenth revisions of *Classification of Diseases Clinical Modification and Related Health Problems* [ICD-9-CM or ICD-10-CM]) for both drug dependence and endocarditis (Supplemental Table; https://stacks.cdc.gov/view/cdc/45932) were included in this analysis. Drug dependence was defined as discharge diagnoses indicating drug withdrawal or overdose/poisoning from or dependence on any drug, including cocaine, opioids, amphetamines, or hallucinogens. Endocarditis outcomes were determined using diagnosis codes for acute or chronic endocarditis, and persons with diagnosis codes suggesting coinfections with HIV or HCV were identified.

Payer status was categorized as private insurance, Medicaid, Medicare, unidentified payer, and other. Patients with unidentified payers included those listed as self-pay (e.g., uninsured), unknown, or missing. Cost was reported as the total cost billed by the hospital. Open-source, state-specific data were used to categorize counties as rural (<250 persons per square mile [ppsm]), a regional city (250–750 ppsm), or urban (>750 ppsm). To calculate the incidence rates of hospital discharge diagnoses for drug dependence combined with endocarditis among the general North Carolina population, census estimates of persons aged ≥18 years for 2010–2015 were used for denominators. Wilcoxon rank sum tests were used to analyze the hospital charge (cost) data. Incidence rate ratios (IRRs) were used to compare incidence rates by age. Analyses were performed using spreadsheet and statistical software.

During 2010–2015, a total of 505 North Carolina residents aged ≥18 years were hospitalized with the two diagnoses of drug dependence and endocarditis (Table). Nearly two thirds of patients were aged ≤40 years, including approximately half who were aged 26–40 years. Patients were mostly white (87%) and non-Hispanic (92%), and the majority (60%) were from rural counties. Nineteen percent of patients hospitalized for endocarditis were uninsured and 23% were on Medicaid. HIV coinfections were uncommon (1.4%), but 36% of patients with endocarditis had past or current HCV infections.

**TABLE:** Characteristics of patients hospitalized with drug dependence and endocarditis (N = 505) — North Carolina, 2010–2015

Characteristic	No. (%)
**Age at hospital admission (yrs)**	
18–25	82 (16)
26–40	245 (49)
41–60	131 (26)
>60	47 (9)
**Gender**	
Male	240 (48)
Female	265 (52)
**Ethnicity**	
Non-Hispanic	465 (92)
Hispanic	7 (1)
Unknown	33 (7)
**Race**	
African-American	41 (8)
White	440 (87)
Other	24 (5)
Geographic classification*	
Rural	302 (60)
Regional city	128 (25)
Urban	75 (15)
**Insurance payer**	
Private	215 (43)
Medicaid	116 (23)
Medicare	67 (13)
Other	10 (2)
Unidentified/Uninsured	97 (19)
**Other infections**	
Hepatitis C virus	181 (36)
Human immunodeficiency virus	7 (1.4)

* Rural = <250 persons per square mile (ppsm); regional city = 250–750 ppsm; urban = >750 ppsm.

The incidence of hospital discharge diagnoses for drug dependence combined with endocarditis among the general North Carolina population sharply increased during the study period, particularly beginning in 2013 (Figure 1). Rates of hospital admissions for drug dependence–associated endocarditis increased approximately twelvefold, from 0.2 cases per 100,000 persons per year in 2010 to 2.7 cases per 100,000 persons in 2015. The sharpest rate of increase occurred among persons aged 18–25 years (IRR 2.1; 95% confidence intervals [CI] = 1.4–3.1) and 26–40 years (IRR 3.8; 95% CI = 2.8–5.1) compared with rates in persons aged >40 years.

**Figure 1:**
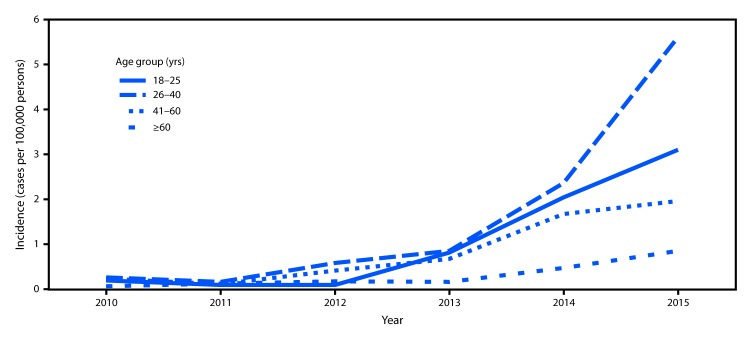
Incidence* of hospital discharge diagnoses of drug dependence–associated endocarditis,^†^ by age group — North Carolina, 2010–2015 * North Carolina Hospital Discharge database, which includes discharge data from all 128 hospitals in North Carolina. ^†^ Ninth and tenth revisions of *International Classification of Diseases Clinical Modification and Related Health Problems* (ICD-9-CM or ICD-10-CM) codes for both drug dependence and endocarditis.

The median hospital charge for drug dependence–associated endocarditis hospitalization was $54,281; total costs of hospitalizations for drug dependence–associated endocarditis increased eighteenfold during 2010–2015, from $1.1 to $22.2 million (Figure 2). In 2015, 42% of patients with drug dependence–associated endocarditis were either uninsured or on Medicaid, accounting for a total $9.3 million in health care costs compared with only $481,000 in 2010 (p<0.01).

**Figure 2:**
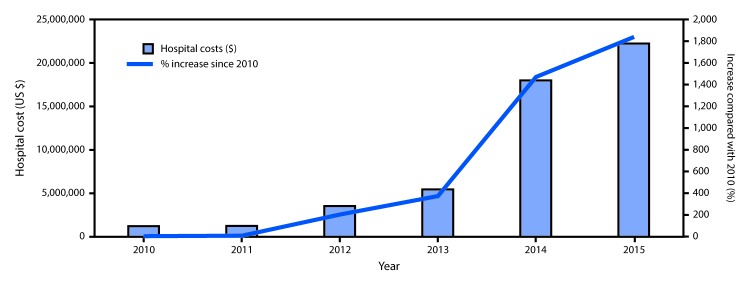
Hospital costs for persons with drug dependence–associated endocarditis, and percentage increase since 2010 — North Carolina, 2010–2015

## Discussion

The incidence of hospitalizations for drug-associated endocarditis is increasing rapidly, particularly among drug users who are younger, white, non-Hispanic, and from rural areas (*7*–*9*). Approximately one third of patients hospitalized with drug dependence–associated endocarditis in North Carolina during 2010–2015 were coinfected with HCV; this finding was not unexpected because injection drug use is a recognized risk factor for both endocarditis and HCV infection (*5*,*7*–*9*).

Among patients hospitalized for drug dependence–associated endocarditis, 42% were uninsured or had Medicaid coverage, suggesting that the health care system and public payers could share a larger proportion of the cost of the increasing incidence of endocarditis, particularly if the costs of infectious complications of injection drug use, including endocarditis and HCV, continue to rise. These findings suggest a need to focus preventive interventions on harm reduction strategies such as syringe service programs, safe injection education, and treatment programs offering opioid agonist and antagonist therapies (*10*).

The findings in this report are subject to at least four limitations. First, ICD-9-CM and ICD-10-CM codes for drug use are subject to coding errors and misclassification (e.g., historic use versus current use). Only hospitalizations with drug dependence listed as a diagnosis were included in this analysis, but patients might not have disclosed drug use; thus, hospitalizations for drug dependence–associated endocarditis might have been under-ascertained. Second, administrative codes do not provide sufficient information to identify a causal association between current drug use and developing endocarditis. Third, administrative codes are nonspecific and do not identify the mode of drug dependency (e.g., injection, oral, or inhalation). Finally, the charge data do not reflect the actual cost to the health system or to the payer, but rather the initial charge billed by the hospital. Therefore, the cost data might be overestimated because of insurance-negotiated pricing.

In North Carolina, the incidence of hospitalizations for endocarditis among drug-dependent patients has increased twelvefold since 2010. Simple and cost-effective public health interventions such as syringe service programs and harm reduction strategies that include the use of fact-based drug education, drug-related illness and injury prevention, and drug treatment could lead to decreased morbidity as well as potential cost savings for the health care system in North Carolina. Coordination among public health providers, health care systems, and policy makers is essential to address the growing U.S. opioid epidemic and its consequences.

**Summary**

**What is already known about this topic?**

Injection drug use and opioid dependence have increased to epidemic levels in the United States, and evidence suggests that bacterial complications of injection drug use, such as endocarditis, are increasing.

**What is added by this report?**

In North Carolina, analysis of hospital discharge data identified an approximately twelvefold increase in hospitalizations for endocarditis combined with drug dependence during 2010–2015. Consistent with overall trends in the U.S. opioid epidemic, the majority of patients were non-Hispanic, white, aged <40 years, and from rural areas; in addition, approximately one third were infected with hepatitis C virus. On average, the cost for each hospitalization for endocarditis exceeded $50,000, and 42% of hospitalizations were among persons on Medicaid or without insurance. The total hospital costs of hospitalizations for drug dependence–associated endocarditis increased eighteenfold during 2010–2015.

**What are the implications for public health practice?**

As the U.S. opioid epidemic continues to grow, hospitalizations for infectious complications associated with injection drug use are likely to increase. Effective and cost-saving public health interventions, such as syringe service programs and harm reduction strategies, are needed to reduce disease burden and save health care costs. Collaboration between public health, health care systems, and policy makers is important to reduce the risks associated with injection drug use.

## References

[1] Center for Behavioral Health Statistics and Quality. Behavioral health trends in the United States: results from the 2014 National Survey on Drug Use and Health. HHS publication No. SMA 15–4927. Rockville, MD: US Department of Health and Human Services, Substance Abuse and Mental Health Services Administration, Center for Behavioral Health Statistics and Quality; 2015. https://www.samhsa.gov/data/sites/default/files/NSDUH-FRR1-2014/NSDUH-FRR1-2014.pdf

[2] Jones CM. Heroin use and heroin use risk behaviors among nonmedical users of prescription opioid pain relievers—United States, 2002–2004 and 2008–2010. Drug Alcohol Depend 2013;132:95–100. 10.1016/j.drugalcdep.2013.01.007 23410617

[3] Rudd RA, Aleshire N, Zibbell JE, Gladden RM. Increases in drug and opioid overdose deaths—United States, 2000–2014. MMWR Morb Mortal Wkly Rep 2016;64:1378–82. 10.15585/mmwr.mm6450a3 26720857

[4] Scheidegger C, Zimmerli W. Incidence and spectrum of severe medical complications among hospitalized HIV-seronegative and HIV-seropositive narcotic drug users. AIDS 1996;10:1407–14. 10.1097/00002030-199610000-00014 8902071

[5] Klevens RM, Hu DJ, Jiles R, Holmberg SD. Evolving epidemiology of hepatitis C virus in the United States. Clin Infect Dis 2012;55(Suppl 1):S3–9. 10.1093/cid/cis393 22715211 PMC5774980

[6] Wilson LE, Thomas DL, Astemborski J, Freedman TL, Vlahov D. Prospective study of infective endocarditis among injection drug users. J Infect Dis 2002;185:1761–6. 10.1086/340827 12085322

[7] Ronan MV, Herzig SJ. Hospitalizations related to opioid abuse/dependence and associated serious infections increased sharply, 2002–12. Health Aff (Millwood) 2016;35:832–7. 10.1377/hlthaff.2015.1424 27140989 PMC5240777

[8] Wurcel AG, Anderson JE, Chui KK, Increasing infectious endocarditis admissions among young people who inject drugs. Open Forum Infect Dis 2016;3:ofw157. 10.1093/ofid/ofw157 PMC5084714 27800528

[9] Hartman L, Barnes E, Bachmann L, Schafer K, Lovato J, Files DC. Opiate injection-associated infective endocarditis in the southeastern United States. Am J Med Sci 2016;352:603–8. 10.1016/j.amjms.2016.08.010 27916215 PMC5830130

[10] Marshall BDL, Green TC, Yedinak JL, Hadland SE. Harm reduction for young people who use prescription opioids extra-medically: obstacles and opportunities. Int J Drug Policy 2016;31:25–31. 10.1016/j.drugpo.2016.01.022 26919826 PMC4975034

